# Supplementary material for: Maptcha: an efficient parallel workflow for hybrid genome scaffolding
Source: BMC Bioinformatics. 2024 Aug 8;25:263. doi: 10.1186/s12859-024-05878-4 (PMC11313021; doi:10.1186/s12859-024-05878-4)
Supplement: Supplementary file 1 — Supplementary Material 1. [file 12859_2024_5878_MOESM1_ESM.pdf]

Supplementary Material

Benchmark construction for evaluating the mapping step

In our investigation of mapping quality against contemporary benchmarks, we utilized the `Minimap2` tool, a widely recognized tool for read-to-reference mapping, extensively employed in recent hybrid scaffolding methodologies.

For each input in our test data set, we constructed a benchmark to evaluate the longread-to-contig mapping in our scaffolding workflow. These benchmarks were constructed using their mapping information on the reference genome G. Figure S1 shows an illustrative example, involving 6 longreads and 7 contigs. More specifically, to determine the $\langle$start,end$\rangle$ benchmark coordinates of each contig and of each longread, we mapped the set of contigs and the set of longreads to the reference genome using `Minimap2`. After this mapping, we say a longread $r \in \mathcal{L}$ *maps* to a contig $c \in \mathcal{C}$ if and only if their respective coordinates intersect in at least one position of the reference genome. Using this information, we constructed a set of benchmark (for each input): the mapping benchmark consists of tuples of the form $\langle$contig, long read ID$\rangle$;

To compare a test output to the corresponding benchmark, we compute the following measures: a) *True Positive (TP):* when a test output matches with the benchmark; b) *False Positive (FP):* when a test output is *not* in the benchmark; and c) *False Negative (FN):* when a benchmark output is *not* in the test output. Consequently, precision is given by $\frac{TP}{TP+FP}$, and recall by $\frac{TP}{TP+FN}$.

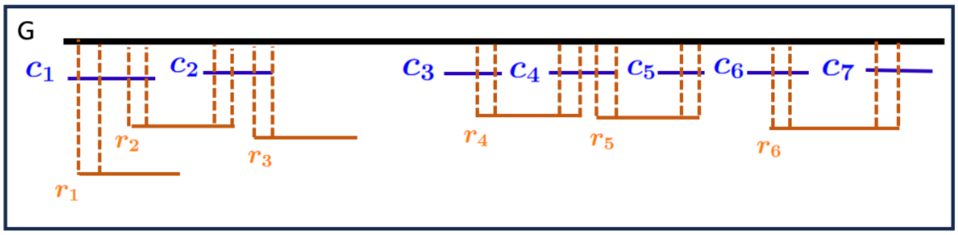

Fig. S1: An illustrative example for constructing benchmarks for evaluation of the mapping, using 7 contigs and 6 longreads. The mapping benchmark for this example will contain tuples $\{\langle c_1, r_1\rangle, \langle c_1, r_2\rangle, \langle c_2, r_2\rangle, \langle c_2, r_3\rangle, \langle c_3, r_4\rangle, \langle c_4, r_4\rangle, \langle c_4, r_5\rangle, \langle c_5, r_5\rangle \ \langle c_6, r_6\rangle \ \langle c_7, r_6\rangle\}$. Consequently, this will contribute to the following edges to the contig graph benchmark: $\{\langle c_1, c_2\rangle$, $\langle c_3, c_4\rangle$, $\langle c_4, c_5\rangle$, $\langle c_6, c_7\rangle\}$.

Mapping quality

The mapping quality of `JEM-mapper` alongside the quality achieved by `Minimap2` is shown in Figure S2. We can observe that the recall achieved by `JEM-mapper` mapping is significantly higher than using `Minimap2`. In all the cases it is well more than the recall rate achieved by `Minimap2`. As for precision, `Minimap2` is better for some inputs

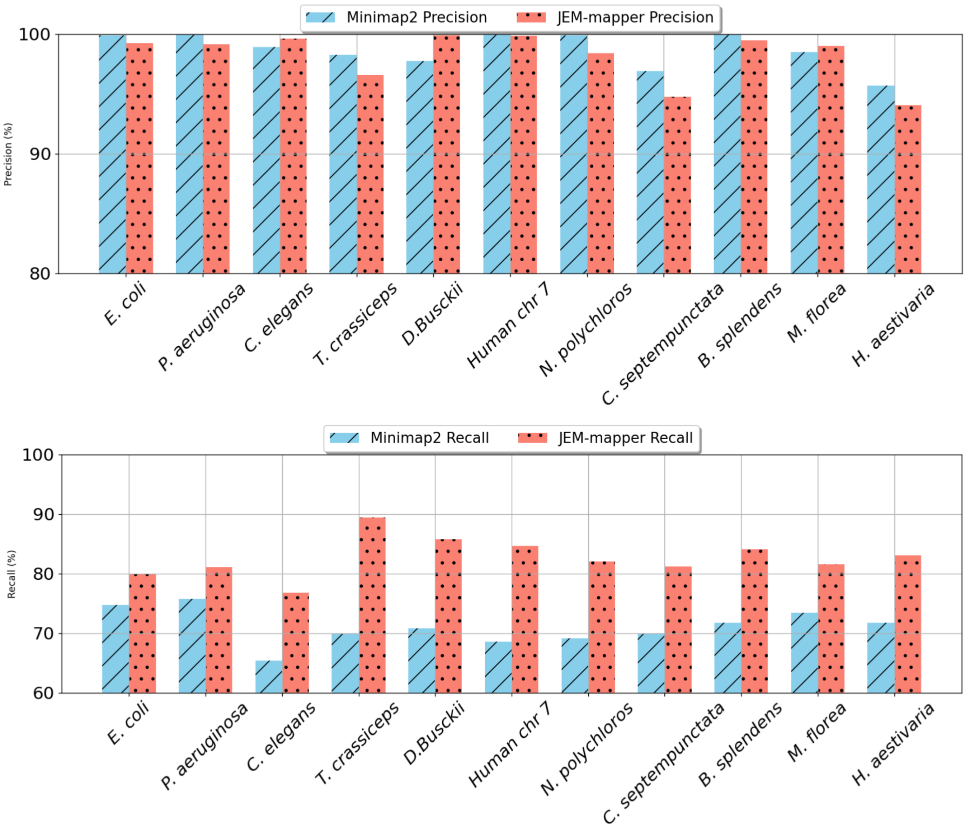

Fig. S2: Precision and recall values for the mapping quality comparison between `Minimap2` and `JEM-mapper`.

like the smaller genomes, *E. coli* and *P. aeruginosa* but `JEM-mapper` is comparable in all cases and higher for some bigger inputs like *C. elegans* and *D. busckii*. Collectively, these results suggest that the MinHash-based scheme holds promise for application in erroneous long read use-cases. The randomness in the procedure to pick the sketches improves recall. This also ensures that there will be substantial contig linking information passed on to the scaffolding step. As for `Minimap2`, the loss in recall can be attributed to the length divergences of the long reads. In particular, we observed that `Minimap2` fails when the length of the contig grows comparable or larger to the long read length; while the stochastic nature of selecting short sketches for `JEM-mapper` coupled with our choice of using only end segments from the longreads, is able to yield better recall rate across the longread length distribution.

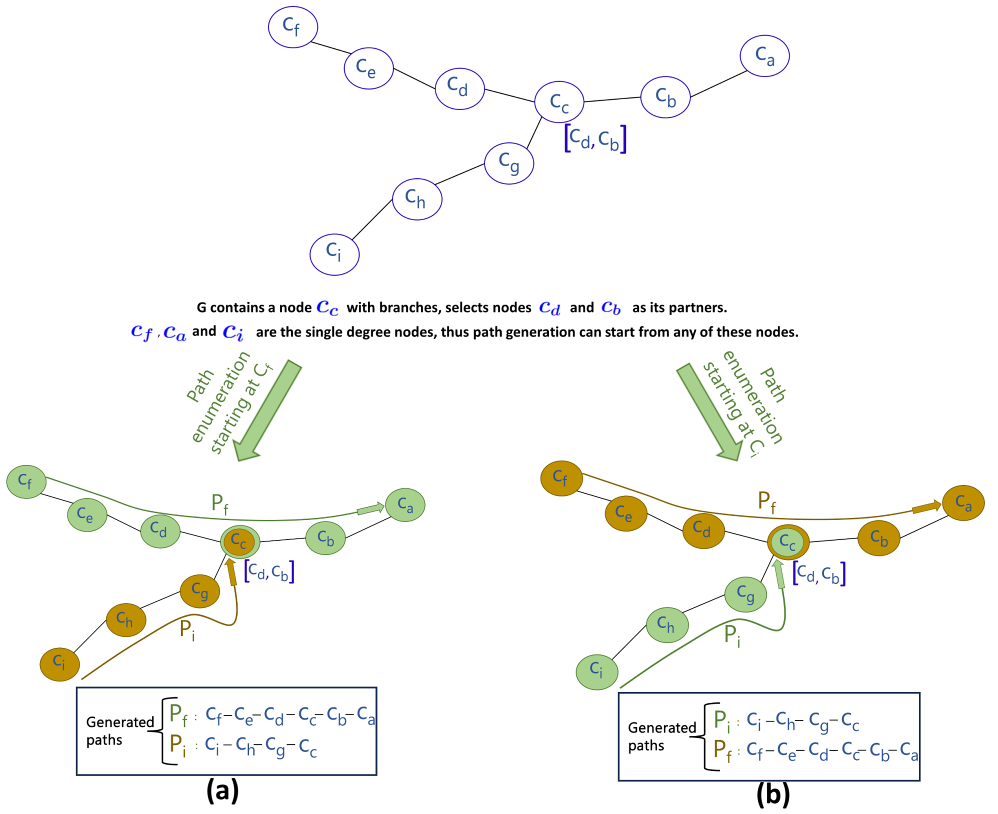

Fig. S3: An illustrative example demonstrating the deterministic routing regardless of the traversal origin. This diagram illustrates a scenario where a network node (c_c) with branching options (c_d and c_b) generates two distinct paths (P_f and P_i) from different single-degree node origins (c_f and c_i, respectively). Path P_f, originating from node c_f, traverses through nodes c_e, c_d, c_c, c_b, and c_a, while path P_i, initiated from node c_i, progresses through nodes c_h, c_g and c_c. Despite different starting points, the same two paths are output, showing the deterministic routing property of the path enumeration algorithm.

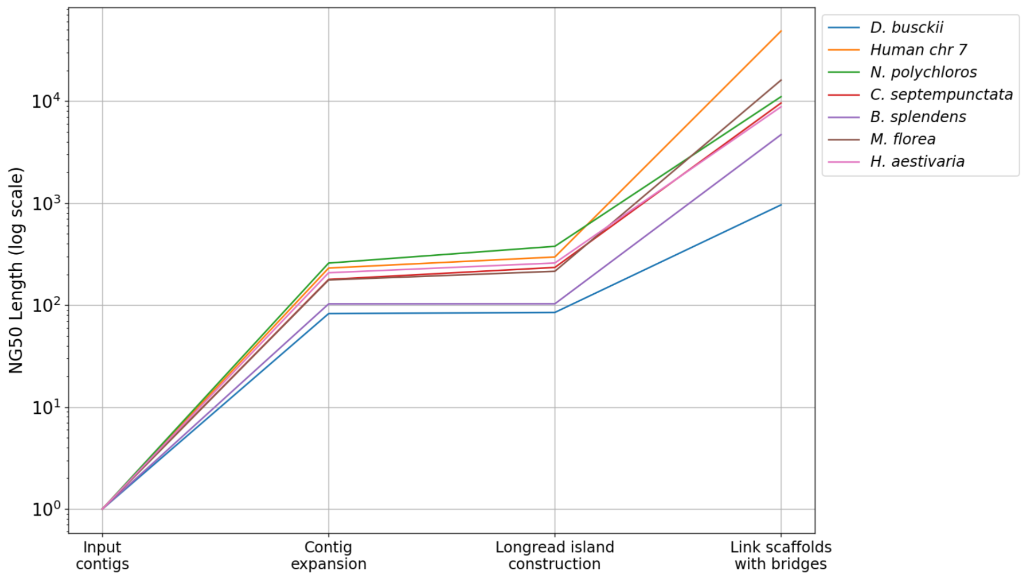

Fig. S4: Qualitative improvement of the partial scaffolds (measured in the NG50 lengths) over the different phases of `Maptcha`. All values (y-axis) are normalized treating the initial contig NG50 length as 1.0 for the respective inputs.

Parameters used:

JEM-mapper was used with the command:

```
mpiexec -np 64 ./jem -s contigs.fasta -q long_reads.fasta \
-a ~/A.txt -b ~/B.txt -p ~/Prime.txt -r 2000 -n 100
```

The command used for LRScaf (v1.1.12) is:

```
java -jar "~/LRScaf/LRScaf-1.1.12.jar" -c contigs.fa \
-a alignment.mm -t mm -t 64 -o ~/LRScaf/Output/
```

The command used for ntLink (v1.3.10) is:

```
ntLink scaffold target=contigs.fa reads=longreads.fa k=32 w=250 \
t=64 v=1
```

Input genome	Method	# N's (per 100 kbp)
E. coli	LRScaf	2,603.64
	ntLink	3,569.83
	Maptcha	0
P. aeruginosa	LRScaf	1,881.45
	ntLink	2,847.15
	Maptcha	0
C. elegans	LRScaf	12,250.60
	ntLink	19,823.88
	Maptcha	0
T. crassiceps	LRScaf	1,116.73
	ntLink	4,889.74
	Maptcha	132.46
D. busckii	LRScaf	4,489.79
	ntLink	11,018.74
	Maptcha	848.47
Human chr 7	LRScaf	3,918.50
	ntLink	30,433.43
	Maptcha	94.81
N. polychloros	LRScaf	–
	ntLink	38,069.68
	Maptcha	0.53
C. septempunctata	LRScaf	–
	ntLink	45,173.33
	Maptcha	5.98
B. splendens	LRScaf	–
	ntLink	13,244.20
	Maptcha	6.78
M. florea	LRScaf	–
	ntLink	42,544.76
	Maptcha	4.87
H. aestivaria	LRScaf	–
	ntLink	52,064.40
	Maptcha	11.61

Table S1: Number of Gaps (N's) per 100 Kbp for all the output scaffolds. All statistics shown are for the final output scaffolds. Symbol – indicates that the corresponding runs did not complete within 6 hours

Input	GenBank assembly ID	NCBI BioSample ID
E. coli	GCA_000005845.2	SAMN02604091
P. aeruginosa	GCF_000006765.1	SAMN02603714
C. elegans	GCA_020450165.1	SAMN21536911
T. crassiceps	GCA_023375655.1	SAMN25949382
D. busckii	GCA_011750605.1	SAMN13899428
Human chr 7	GCA_000001405.29	PRJNA31257
N. polychloros	GCA_905220585.2	SAMEA7523477
C. septempunctata	GCA_907165205.1	SAMEA7520205
B. splendens	GCA_024678985.1	SAMN14784700
M. florea	GCA_930367185.1	SAMEA7520156
H. aestivaria	GCA_947507615.1	SAMEA7701444
H. magnus (draft assembly)	GCA_026573805.1	SAMN20982429
H. magnus (long reads)	SRR15840267	–

Table S2: Accession IDs for all reference genome builds used for the simulated test inputs and the real-world dataset. Symbol – signifies no BioSample ID was available.
